# A qualitative exploration of behavioral factors affecting mothers of malnourished children under 5 years old in Kiribati

**DOI:** 10.12688/f1000research.17732.2

**Published:** 2019-04-09

**Authors:** Antje Reiher, Masoud Mohammadnezhad

**Affiliations:** 1 Department of Public Health, Ministry of Health and Medical Services, Nawerewere, Tarawa, Kiribati; 2 Department of Public Health, Fiji National University, Suva, Fiji

**Keywords:** Mothers, Malnutrition, Children under 5 years, Qualitative study, Kiribati

## Abstract

**Background: **In Kiribati, malnutrition is the leading cause of death for children aged less than 5 years. The purpose of this study was to explore contributing behavioral factors among mothers of malnourished children under 5 years old in Kiribati.

**Methods: **This qualitative study was conducted in an urban area of South Tarawa among mothers of malnourished children aged less than 5 years in 11 public health centers in 2016. The study included 9 focus group discussions, with a sub sample of 3 to 4 in each group, having a total of 35 participants. Using a semi-structured questionnaire, data was collected and thematic analysis was applied to analysis the data.

**Results: **Seven main themes were identified including; knowledge, behaviors, perceived severity, perceived benefits to action, perceived barriers and cultural related issues. These encompassed a variety of reasons which could explain the malnutrition in children of those particular mothers.

**Conclusion: **In order to tackle malnutrition in Kiribati, it is crucial to identify the main factors that are hindering this preventable disease. This study provides information essential to enhanced decision making, health care delivery planning and has policy implications for the improvement of quality of health care in Kiribati.

## Introduction

Malnutrition remains a major problem not only in developing countries, but worldwide affecting mostly children under 5 years old ^1, 2^. The first 1,000 days of the child’s life affected by poor nutrition can lead to stunted growth, which is a permanent result and is related with impaired cognitive ability and reduced school and work performance ^2– 6^. The consequences of malnutrition are varied and include: increased susceptibility to infection, impaired child development, increased mortality rate and individuals will function in substandard ways ^7, 8^. The World Health Organization (WHO) revealed that globally, childhood malnutrition accounts for approximately 35% of all deaths among children under the age of 5 years ^9^. A report on malnutrition has stated that severe malnourished children have a higher risk of death from ordinary childhood illness such as diarrhea, pneumonia ^10^.

Malnutrition amongst children under 5 years old is a major public health issue, particularly on the main island of South Tarawa in the Republic of Kiribati with nearly 15% of the children underweight and with considerable gaps in immunization ^11^. Globally, Kiribati contributed to 28% of all under 5 deaths, with 15% of under 5 year old due to severe malnutrition ^12^.

United Nations Children’s Fund (UNICEF) statistics show that the total population of Kiribati under 5 was 11,000, of which there are 6,934 children aged 0–4 on South Tarawa alone - this is almost 14% of the total population ^13^. Kiribati faces challenges to improve its nation’s health-care as malnutrition and diarrhea remain major problems ^11^. The 2009 Demographic health survey reported 23% of children are underweight or severely underweight ^14^. This figure positioned Kiribati above the WHO threshold (10%), making the prevalence of underweight children a significant public health issue. More children are underweight amongst the poorer households than in wealthier ones ^15^. Therefore, it is important for the health system to detect malnutrition at an early stage for planning and implementing timely interventions at the community level.

The role of culture in Kiribati is very important as it can affect malnutrition in children. For example, people living in extended families, where one family accepts people from their extended family to come and stay in one home and eat together. Local tradition that everyone in the household should be fed shows that limited resources are distributed between members in the extended family, plus unexpected visitors ^16^. In this culture, elders are highly respected people in the family. They will be provided better food, so it can be seen as a barrier to the use of appropriate practices for feeding infant and young child.

Lloyd stated that globally, mothers are charged with the task of feeding and providing care for their children, irrespective of the environment or resources available to them ^17^. It is important to recognize that many interventions to improve child health and nutritional status depend on someone’s behavior, frequently the mother ^18^. It is vital to study the knowledge, attitude and practice (KAP) of mothers regarding breastfeeding, complementary feeding, dietary practices and immunization, as these factors have a huge impact on young children’s health. Therefore, mothers as the primary caretakers for young children need to be knowledgeable in looking after their children, especially earlier in life.

Consequently, this study aims to identify the KAP of mothers of malnourished children (under 5 years old) with respect to behavioral contributing factors to malnutrition (breastfeeding, complementary feeding, dietary practice and immunization) on South Tarawa in Kiribati.

## Methods

### Study design and setting

A qualitative study design was conducted for almost 8 weeks from 21 ^st^ of December 2015 to 12 ^th^ of February 2016, to determine the level of mother’s KAP on breastfeeding, weaning, dietary and immunization on South Tarawa (capital island of Kiribati). Tarawa is the capital island of Kiribati and is divided into two parts, North and South Tarawa, with South Tarawa being the only part of Kiribati considered ‘urban’. The focus group discussion (FGD) method was used which allowed participants to discuss problems in a group setting and allowed more detailed responses.

The FGD was held at nine different public health clinics on South Tarawa during working hours, as ‘saturation’ was reached after nine FGDs, meaning there was no new information or emerging themes ^19^.

### Study population: inclusion and exclusion criteria

The inclusion criteria were all women with children under 5 years old who were identified as being malnourished and were admitted to the Pediatric wards of selected public health clinics within the period of 1 ^st^ January to 31 ^st^ May 2015. The study participants had to be Kiribati citizens (self-identified) and their children’s names must be registered under the public health clinics. The exclusion criteria were mothers who didn’t meet the study inclusion criteria and were not willing to participate in the FGD, mothers or children who were not residents on South Tarawa and mothers with children aged more than 5 years old. Critical stage children that were admitted to the Pediatric ward were also not included.

### Sampling, sample size and data collection tools

A non-probability purposive sampling method was used to recruit participants for nine FGDs. Each group contained 3 to 4 participants, with a total sample size of 35 participating mothers.

A semi-structured open ended questionnaire was used in this study whereby there were two open-ended questions allocated for each of the factors (breastfeeding, complementary feeding, dietary practice and immunization). Prior to collecting the data, the questionnaire was validated using two group discussions, during child health care visits. The participants who met the inclusion criteria were selected and given the questionnaire to answer to ensure the questions were understandable and readable for face validity. Content validity was also used whereby three academic experts (assistant professors and lecturers at the Public Health at Fiji National University) assessed the construction in question and checked whether the responses by the person answering the questions were affected by other factors. Once done, their comments were revised accordingly and a new version of the questionnaire was then distributed among the participants.

### Data collection process

Mothers that were interested to participate in the study then signed a consent form to proceed with the discussion. The places and time of the discussions were chosen by the researcher that was convenient to the participants. All participants sat together in a private room in each public health clinic, in a circle, so the main researcher could have equal access with the voice recording. The researcher, who had previous experiences in conducting FGDs, and was bilingual, was present and available to translate the questions from English to Kiribati language for participants to have a clear understanding of the questions raised during discussion. The discussions were in the local language (Kiribati) and both audio recording and note taking were used.

The discussion began informally with the greetings and personal instructions from the researcher. When all participants were settled and ready to start, the researcher led the discussion by starting with a question for each topic on breastfeeding, complementary feeding, dietary practice and immunization. The participants in each group mingled well with each other so they communicated freely with one another. Each FGD had an average duration of 60 minutes. All conversations during the FGDs were conducted in Kiribati language so that participants were able to talk freely without any restrictions in expressing their opinions.

### Semi-structured questionnaire

Questions were as follows:

Breastfeeding:

1.   What are your perceptions on breastfeeding?

2.   What problems you faced while breastfeeding and how did you overcome it?

Weaning:

1.   How do you know it is right to start weaning and how do you approach it?

2.   What foods do you give your baby when weaning, home-made foods or store-bought foods and why?

Diet:

1.   What are the obstacles you (mothers) you face in making recommended diet for your children?

2.   As your baby gets older what do you think will be the key things that you will be concerned about diet wise?

Immunization:

1.   What are your perceptions on immunization? Why are children immunized?

2.   What are obstacles to immunizing your children and why?

### Data management and analysis

The researcher transcribed all digital recordings of the focus groups in Kiribati language, then translated these transcripts into English language. Transcripts and field notes were compared to ensure that no information was misinterpreted or omitted.

Data were then analyzed manually, by the author, to discover inter-rater coding dependability. After the first review of the data, a categorizing method was developed by using thematic analysis based on the study objectives. Then the researcher identified themes and sub-themes developed from the data analysis and lastly produced a final thematic index to code the data. The researcher listened to the recordings and read the written notes numerous times to understand the participants’ views and ideas they expressed through their own words. Data were then interpreted and conclusions were drawn based on coding summaries and contextual field notes provided by direct quotes from participants. Codes were then grouped into themes which can be defined as repeated features or patterns.

### Ethical considerations

The ethical approval for this study was received from the Kiribati Ministry of Health after the research proposal was approved by Fiji National University’s College Health Research Ethics Committee (CHREC).

## Results

### Socio-demographic characteristic of the participants

The majority of the women in the study were aged 19–32 years, followed by 26–32 years. Most of the participants had attended high school (67.1%) and the majority of them were living in a family with 5–9 people (46.3%). More than two thirds of participants (85%) were married and 31.7% of mothers had only 1 child.

Seven main themes were identified: knowledge, behaviors, perceived severity, perceived benefit to action, perceived barriers, cues to action and cultural related issues.

### 1. Knowledge

Knowledge and understanding from experiences is another way to carry out things in our daily lives. When mothers were asked about when was the right time to start complementary feeding, only two groups mentioned that complementary feeding should be started when the child is refusing to breastfeed.

*I start complementary feeding because my child refuses to breastfeed and crying most of time and when I start feeding him I knew that he want to eat more than to breastfeed, so I think that is a good sign for me to start giving complementary food to my child. (FGD 5).*

Four different groups were not sure about the right age to start complementary feeding. One mother from one group said that 1 year old is the right age to start complementary feeding, whereas others said it was 2 years old, 3 years old and 4 years old.

Mothers provided different ranges of knowledge when asked about their perception on breastfeeding. Most of the groups (6 groups) stated that breastfeeding will prevent disease to their child and make the child healthier (5 groups). One participant said that during antenatal and postnatal clinic, she always got advised by the nurses about the importance of breastfeeding;

*I always breastfeed all my children because I know it is good to prevent them from disease but also because I do not want to risk my child life and to blame myself for it as I already know the importance of breastfeeding from the nurse. (FGD 4)*

When mothers were asked about their perception on immunization, a few of the groups (4 groups) stated that immunization was free of charge. One participant mentioned that immunization is important to her child and it is a service that is provided so you do not have to spend money on it:

*I understand that immunization does not cost any money and nurses were willing to visit us at home when you did not come to take our child for immunization. It is the best opportunity for our children and we supposed to make use of it. (FGD 9)*

Mothers had different knowledge when asked about the key things on diet when their child becomes older. Most of the groups (7 groups) reported that more solid food and a balanced diet should be introduced when their child is older. They are more knowledgeable on the types of food to give their children because they were given a pamphlet during the child health clinic to guide them with feeding their child and from their past experiences from previous children. One mother pointed out that nurses play a major role in assisting mothers to know how to provide the proper food to their children:

*I am very thankful that nurses are very concern about our children and to make sure we are giving them the right food. Health education they give us with pamphlet to take home is of good benefit to our children but it is then over to us mothers on how to practice this at home is a different story. (FGD 3)*

### 2. Behaviors

Many of the FDGs reported that forgetfulness was continuously the reason for not immunizing their children on schedule. This was because they were not concerned about the effect of not being immunized would have on the children. They thought it was alright to miss their child’s vaccines and when they remember next time they will go and attend immunization clinic.

*Sometimes I forgot to take my child for injection because when I was given an appointment card and when I get home I just leave it lying anywhere and then I forgot the time to come back to the clinic. I will find another time that suit me to go to the clinic and take my child for injection when I remember. (FGD 1)*

Mothers had different behaviors when asked about breastfeeding. Most groups (5 groups) stated when they were faced with breast milk insufficiency the only option is to give formula milk straight away. One participant claims that because she does not want her child to get hungry and cry she had no choice but to give formula milk.

*Even though I understand the importance of breastfeeding, I cannot stand to see my child crying and because I understand that there is a formula milk sold for babies, my only option is to buy it and give to my baby. (FGD 8)*

In regards to immunization, most of the mothers (7 groups) had poor behaviors in getting their child immunized with forgetfulness as one of their obstacles in fulfilling a good immunization record. One participant admitted that forgetfulness is a common excuse they will provide for not attending to their child immunization.

*I am always worried of what to say to the nurse the next time I visited the clinic for my child immunization, but because I was not ready to take my child for immunization at the certain booked times. I always make excuse and tell the nurse that I forgot about the immunization appointment given to me. I experienced that the only way to stop the nurse from getting upset with us for not attending immunization is forgetfulness. (FGD 3)*

### 3. Perceived severity of health issue (malnutrition)

When mothers responded to a question on what obstacles they faced in immunizing their children, most of the focus groups (5 groups) stated that mothers worried that their child will get a fever after receiving an immunization. One mother explained that they do not want to take their child for immunization because they feel sorry for their child and they do not want to stay up and cool sponge the child when he/she gets a fever:

*I do not mind taking my child for injection because I know that it is important for him but the problem is that I am not accepting the facts that I do not have to sleep well and look after my child when he gets a fever. (FGD 2)*

### 4. Perceived benefits of action

Out of the nine FGDs, most groups (6 groups) believed that breastfeeding prevented disease in the child. Most mothers continue to breastfeed their children because they are advised during antenatal and postnatal clinics that breastfeeding is important for their children because it will protect them from infectious diseases. They had the knowledge that breastfeeding is critical, especially during their child’s young age. In one group, the mother shared her experience and compared her situation with one of her siblings that did not breastfeed her children.

*Breastfeeding is good for the child because it protecting the child from infectious disease. I experienced this because my younger sister never breastfeed all her two children and these children are always sick when there is an outbreak of diarrhea they get it and having a recurrent episode of pneumonia as well. I feel sorry for these children. (FGD 1)*

When they were asked about their perception on immunization and why their child is immunized, all nine FGDs mentioned that immunization is preventing disease to young children. However, mothers had problems getting the child to their appointment for immunization because they were too lazy to walk to the health clinic. They are expecting the nurse to visit them at home to give immunization injections. One of the mothers in a group explained that they continue to immunize their child because they know the importance and benefits of it to their children.

*I just knew that after the completion of our injection, I just understand that the injection really protected them from getting the diseases. But the big issue is how to get there, that is why we cannot complete our child’s injection on schedule. I think it is related with our poor attitude of laziness to get to a far place. (FGD 3)*
*Children are giving injection to make a child healthy and prevent them from disease and also prevent them from getting blindness. (FGD 6)*
*Immunization is good for my child because it prevents disease and also when your child get the disease it not as critical and it just hit your child and gone. (FG5)*

In responding to the question about their perception on breastfeeding, four focus groups said that breastfeeding is convenient and enhances the child’s learning ability. They explained that breast milk, food for their baby, is always available at any time, and they do not have to do much work to prepare it for their child. Mothers also mentioned that breastfeeding enhances the child’s learning ability, as it is good for the child’s brain. They understand that a breastfed child is more intelligent in school compared to someone that was not breastfed. One participant stated that when she goes somewhere with her baby she does not have to worry about taking a lot of things as breast milk is always available and convenient:

*I really agree that breastfeeding is available and convenient for our baby, I do not like carrying so many things plus my child when I went to visit my families, that is why I always breastfeed all my children. (FGD 3)*

### 5. Perceived barriers to action

Among the nine FGDs it was found that there were barriers to action, such as one’s belief in the physical and psychological costs of the advised behavior. When mothers were asked about problems they faced while breastfeeding many groups reported that they were having problems with breast milk insufficiency. One participant said that she is not getting enough breast milk when she breastfed her child and she got advice from the nurse to start giving formula milk while continuing with breastfeeding.

*I gave birth in the hospital and my breast milk is not enough so the nurse advised me to give formula milk and to continue with to breast feed milk because my child is born prematurely. (FGD 7)*

Two focus groups reported that breast abscess, the child refusing, and no breast milk were problems they faced while breastfeeding. One participant mentioned that she had a breast abscess in one breast and she knew that she was not giving as much breast milk to her child as when she was giving both breasts to feed her child.

*I did not breastfeed my child well because I had breast abscess so only 1 breast to give to my child, this always happens when I got pregnant. Therefore, I knew that I did not give enough breast milk to my child, but I keep breastfeed with only 1 breast. (FGD 5)*

Most of the mothers (6 groups) mentioned that breast milk insufficiency is another barrier they are facing with breastfeeding. One participant from two different groups stated that they did not have breast milk at all after delivery and they felt bad for their child because she was always crying.

*I am not sure what happen but I keep breastfeeding my child after birth and I was unable to produce milk, my child is always crying and my mother give me local medicine to get the breast milk out but still not coming out, so I started to give formula milk as I feel sorry for my baby. (FGD 7)*

Mothers are facing obstacles to immunizing their children. A few of the groups indicated that these obstacles included a family problem, loss of their child immunization card and moving to a new place. They stated that when they are fighting or arguing with their husband it affects everything and they are not prioritizing what is best for their child. Another view is that when they lose their immunization card, they are scared to attend immunization clinic as the nurse will scold them for losing an important card so they stay home. When mothers move to a new place and they are not sure where to take their child for immunizations, they miss taking them for immunization.

*There is one tough old nurse in the health clinic that everyone is always scared of. One time I lost my child immunization card and she told me off that I cannot take responsibility and look after my child cards and from that time I felt embarrassed because she scolded at me in front of other people so I prefer to stay home with my child after that. (FGD 9)*

The public health nurses’ poor attitude contributed a lot to the barriers for mothers to attend immunization clinics. Two different responses from mothers said that once they miss their child immunization they will not go again. This happens because nurses are not welcoming or encouraging mothers to attend a regular immunization clinic. One participant said that nurses should be politer when talking to mothers because they are causing mothers to be reluctant about getting their child’s immunizations.

*I wish to have a good nurse in our clinic that can accept our failures and encourage us to bring our child to immunization clinic more often without any restrictions. (FGD 3)*

### 6. Cues to action

One participant had stated she missed taking her child for immunization because of advice from other people, especially old people. She was told that there is no point of getting immunizations because the other generations did not receive any immunization and they are still healthy and surviving till now.

*I was told from my grandfather that they is no such thing as immunization, and I should not be taking my child for immunization. It is giving lots of bad things to the child such as pain, fever and there is no use. (FGD 2)*

In responding to a question about “what are the obstacles they are facing in making a recommended diet for their children?” most groups (5 groups) stated that they cannot give proper food to their children because of peer group advice. Most mothers said that rumors from other mothers in the community affected the way we feed our children. One participant said they were advised to control the food you give to your child while they are young because when they grow up it is hard to control them.

*I always had this advice in mind that I should give less food to my child and not to feed them lots of protein foods because they will grow fat in their young age and it will not be healthy for them when they get older. Therefore, I tried to limit the food I give to my children because of the advice from other mothers. (FGD 3)*

### 7. Culture-related issues

According to the mothers’ FGD, five groups stated that children should be given complementary foods when the mother is pregnant again or if the child is having diarrhea after breastfeeding. It is common that when the mother is pregnant the grandmother makes sure the child is not breastfed again. One of the mothers shared her experience with her child when she got pregnant again.

*I started giving complementary feeding to my child when I fall pregnant again and my mother advised me to stop breastfeeding otherwise my child is weak or not as active. I knew it is our culture so she took my child away from every time she saw me breastfeeding and she is very upset with me, I knew that our mothers are very controlling especially when to do with their grandchildren. (FGD 5)*

Another issue with culture is that people are living in extended families and the appropriate food that should be given to young children was affected by it. When asked about “obstacles faced in making a recommended diet for their children”, three focus groups described that they cannot give special food to their young children because there are so many people living in one home. One of the respondents claimed that they feel sorry for their young children because it is not fair for them to be treated the same way as adults.

*I always feel bad and wish that one time will be stayed alone with our children in our house, but it is hard because we do not want our relatives to think that we are not welcoming them to our home and they might be talking bad about us. It is so hard but our young children are suffering a lot from this. (FGD 4)*

## Discussion

Our study showed that mothers were not aware of a balanced diet and most of them lacked the needed knowledge of what a balanced diet which should be given to their child. To prevent unhealthy weight gain among children it is necessary to reduce total fat to less than 30% of total energy intake ^20^. Mothers need to enhance their knowledge on diet and understand the proper diet or balanced diet to be given to their children and what portions should be given to a grown-up child. It is vital that mothers have a different way of thinking towards the right types of food to give to their children. “A low level of maternal education and a lack of knowledge on good childcare practices means children do not receive optimal nutrition and care” ^21^. Even when mothers do know about the importance of breastfeeding, complementary feeding, balanced diet, and immunization, the lack of progress is more complex. These factors are the role of a mother.

However, health workers should consider that lack of awareness is not the only barrier; habit, socio-cultural constraints, and social norms make it difficult to change ^22^. During child health care clinics, health professionals should conduct health awareness to enhance mother’s knowledge on a healthy, balanced diet through different stages in the child’s life. According to Briscoe and Aboud, in order to change the practice of mothers or caregivers, an active learning approach is considered for the implication that a trained worker needs to demonstrate and caregivers need to practice ^23^. This strategy is more effective than reading information alone.

Mother’s perception of breastfeeding in the present FGDs showed that many of the groups knew breastfeeding was helping to prevent disease and make the child healthier. Nurses were the major sources of information during ante-natal clinic. As mentioned by the WHO, breastfeeding is a natural way of providing young infants with the nutrients they need for healthy growth and development ^24^. The information mothers receive during antenatal care might encourage them to breastfeed their children longer.

The complementary feeding time stated by mothers was when the child refused to breastfeed and the recommended age for complementary feeding varied between 1 to 4 years. It is not recommended to begin weaning before 6 months of age, but it is recommended to begin adding complementary foods at 6 months because breastfeeding alone will no longer fully meet the nutritional needs of the child ^11^. Therefore, the type and timing of weaning foods introduced in an infant’s diet have a great impact on the child’s nutritional status ^25^.

As recommended by the WHO, infants should be exclusively breastfed for the first 6 months of life to achieve optimal growth, development and health ^11^. Subsequently, to meet their changing nutritional requirements, infants should be given nutritionally sufficient and safe complementary foods, while breastfeeding remains for up to 2 years of age or beyond ^24^. In a different study by Berra among 240 mothers of under 5 children residing in Nekemte town, Ethiopia, it is stated that most mothers practice early weaning because of insufficient breast milk ^26^. Mothers had different practices and reasons for weaning, which might indicate that their knowledge on weaning is not adequate and needs to be targeted by health professional awareness programs.

Mothers claimed that immunizations had two perceived severities which cause mothers to not take their child for injection: pain and fever. Mothers had bad experiences after taking their child for immunization when they got swelling at the injection site and the baby cried a lot. These bad experiences from poor practice of health workers contributed to the mother not taking their child for immunization. Health workers should be encouraged and updated on standard procedures to give injections to avoid poor practice as it showed a poor impact on children immunization records. Additionally, health workers, especially in the public health clinic, should reassure mothers about the side effects of immunization and inform them, prior to injection, that the fever will disappear in one or two days and it shows that the immunization given is working properly.

In this present study, mothers’ perceived benefits towards breastfeeding were that they knew and understood that breastfeeding is beneficial to their children. A similar study by Andrade *et al*. was conducted in a health center in South Brazil which stated the reason the mothers comply to vaccination was because it helped prevent the child from getting various diseases ^27^.

When dealing with food, most mothers perceived benefits to homemade foods because of its freshness and being healthy and part of a balanced diet, as they can add their green leaves to it. Their good perception is not related to what they practice at home, though. They may be struggling to get healthy food for their children due to living in extended families with only one or two people being employed. The government could help find ways to involve people with planting green leaves and vegetables for their families, especially the young children for their growth and development. A similar study in rural Africa found that inadequacy of resources is perceived to be another contributing factor to under-nutrition and hence the intervention dealt with resources that were available and any constraint in accessing to them for future planning ^16^.

In a setting such as Kiribati, culture dominates and hence things could be changed in the opposite direction which could be negative or positive. In the present study, the breastfeeding and complementary feeding of children was affected by the Kiribati culture. When the mother is pregnant again the grandmother is involved with breastfeeding cessation and introducing alternative milk to the child. The local thought is that when the mother is pregnant her milk is not good and it makes the child develop diarrhea, from which the child can then become very sick. This is a similar finding to a study by Kakute *et al.,* which identified cultural factors influencing decisions of mothers in feeding their children ^28^.

In a similar study by Abubakar *et al.,* which was conducted in Kilifi District, on the Kenyan coast, a total of ten FGDs were undertaken with eight to ten mothers in each group. The result reported that in all FGDs, mothers were constrained in their ability to provide optimal care due to the social setting in which they raise their children. One common theme mentioned was living in large households where everything was shared with members of the household and those visiting ^16^.

In this study, older people are a higher priority when it comes to food than the younger ones. If there is good food, it is kept aside for the older people to eat; the rest family members only have the option to use the leftover food with low nutritional value which can contribute children’s malnutrition. The thinking is that older people are weak and respected so they offer what is best for them. This cultural practice is common in Kiribati settings and it is a huge contributor to a child’s feeding. These cultures are passed on from generation to generation and it is very hard to change this thinking, but there are ways to try and educate them and slowly, in a manner that they will accept, so that the older people are still shown the necessary respect needed in their culture. The intervention for culture is to involve older people in health talks, not only mothers but also the father and grandparents, as they are the people that need to support the mothers and children. It is best to involve them and have them be part of health promotion and to try and change the way of thinking about certain thing, especially with child’s feedings.

As most of the themes which emerged in this study are related to Health Belief Model (HBM) ^29^, it is advised that the Ministry of Health and health care centers develop an intervention using the elements of HBM which consider mothers’ economic and social context.

## Conclusion

The themes raised from the mother’s responses were knowledge, behaviors, perceived severity, perceived benefits, perceived barriers, cues to action and cultural related issues. It is confirmed from this study that in order to efficiently and effectively decrease the incidence of malnutrition among children residing on South Tarawa, mother’s knowledge need to be enhanced, attitudes need to be changed, as well as developing practices to have a very clear role and understanding on breastfeeding, weaning, diet and immunization. This could be fulfilled with the assistance from health professionals, the ministry of health, community and different stakeholders, for a bright future for the young generation of Kiribati who are the future assets of tomorrow. The cultural related issues mostly had an impact on dietary, as they are not prioritizing children’s feedings as important due to men and old people benefitting more from special food.

Participants recognized the benefits of a healthy diet and the positive effects on children. However, due to their status in the family, work and domestic responsibilities along with limited financial resources, healthy and adequate maternal nutrition is not a priority. Improving education for young adolescent girls is key to improving women’s status in society and in their family. Furthermore, educating women offers them the skills to think freely, be involved in the workforce and practice eating habits that benefit not only themselves, but also their future children.

Health promotion activities should be reinforced at child welfare clinics, health facilities and at community level by considering all determinates such as social/economic and cultural structural variables to improve parents’ knowledge of the recommended infant and child feeding practices.

## Ethical statement

The ethical approval for this study was received from the Kiribati Ministry of Health after the research proposal was approved by Fiji National University’s College Health Research Ethics Committee (CHREC). All participants provided written informed consent to participate in the study.

## Data availability

### Underlying data

As the transcripts of the interviews encompass identifiable information and therefore the data are not openly provided. Limited de-identified transcripts of the interviews performed with the mothers (in Kiribati and English) are available on request from the corresponding author ( masoud.m@fnu.ac.fj).
