# Supplementary material for: A qualitative exploration of behavioral factors affecting mothers of malnourished children under 5 years old in Kiribati
Source: F1000Res. 2019 Apr 9;8:83. Originally published 2019 Jan 21. [Version 2] doi: 10.12688/f1000research.17732.2 (PMC6402073; doi:10.12688/f1000research.17732.2)
Supplement: Peer review history

# Peer review: Reviewer response for version 2

**DOI:** 10.5256/f1000research.20687.r46978

**Published:** 2019-05-07

**Authors:** Diana Arabiat

**Affiliations:** 1 School of Nursing and Midwifery, Edith Cowan University , Perth, WA, Australia

I have read this submission. I believe that I have an appropriate level of expertise to confirm that it is of an acceptable scientific standard.

# Peer review: Reviewer response for version 2

**DOI:** 10.5256/f1000research.20687.r46976

**Published:** 2019-04-30

**Authors:** Sari Andajani

**Affiliations:** 1 School of Public Health and Psychosocial Studies, Auckland University of Technology, Auckland, New Zealand

Happy with the amendment made by authors.

I have read this submission. I believe that I have an appropriate level of expertise to confirm that it is of an acceptable scientific standard.

# Peer review: Reviewer response for version 1

**DOI:** 10.5256/f1000research.19390.r43339

**Published:** 2019-03-04

**Authors:** Diana Arabiat

**Affiliations:** 1 School of Nursing and Midwifery, Edith Cowan University , Perth, WA, Australia

This topic is both timely and relevant, since most of children death takes place in the developing world, and there is tremendous need to educate parents about balanced nutrition in children and when to seek medical help to prevent serious sequelae. Moreover, more information about Kiribati is also welcome in the recent literature, since this society is sorely underrepresented.

 The followings are suggestions for manuscript revision:

**Title** 

 The study topic is more related with ‘ ***Nutritional knowledge, attitude and dietary practices of mothers of malnourished children in Kiribati’,*** so the authors should consider revising the study title.

**Abstract**

 The abstract needs editing and reconstruction, in particular with relation to the study aim listed under background. The authors stated exploring factors contributing to malnourishment in children aged below 5 years old, yet the study did not explore demographic or socio-economic factors in mothers that may contribute to malnourishment in children. I would suggest revising the aim into **exploring nutritional knowledge, attitude and dietary practices of mothers with malnourished children under 5 years old**. The study aim should be listed in a constant way in abstract, methods and discussion. As with regard to the results, I would suggest revising and editing the themes accordingly to the major theme of ***nutritional knowledge, attitude and dietary practices*** and then to include related subthemes (e.g., breastfeeding practices,  complementary feeding’ …etc) under the major themes listed in the study.

**Methods**

 It is unusual to use a semi-structured questionnaire for a qualitative design, so I wonder if the author intended to describe the interview guide used for promoting the FG discussion around dietary practices. If so, this section needs to be revised accordingly. Further information about who facilitate the FG discussion, in what language need to be added.

 In page 4, ‘isolated room’ should be changed into ‘private room’. The section related with semi-structured questionnaire needs to be deleted and an example of the questions used in the interview guide can be included within the text. Questions related with immunization is not directly related with the study aim, yet it can be added as a theme emerged or noticed among mothers of malnourished children.

**Results**

 I found the presenting themes confused and confusing. There is a need first to elaborate on the thematic analysis process used in transcribing the FGS and who and how those themes were generated. This should be added as a heading named ‘Analysis of data’ in the methods section. Another section should be added under results and this should include the socio-demographic characteristic of the sample.

 Second, it is important to refine the major key themes emerged in the data analysis along with the subthemes and selected quotations this can be also presented under a table. Significantly, the authors listed attitudes and practices related with immunisation, yet they did not link it directly with mothers’ knowledge or perception of malnourishment in child. Failing to highlight this relation will risk the overall quality of study.

 Third, findings related with perceived barriers to action can be listed nicely as a subtheme for the major theme of ‘dietary practices’. The authors can refine their findings presentation to list beliefs related with breastmilk insufficiency as a contributing factors that negatively impact child’s health and wellbeing in Kiribati. The authors needs to rewrite and present this section before it can be ready for final publication.

**Discussion**

 The first sentence in the discussion needs editing and I would encourage authors to change “were ignorant” with “they lacked needed knowledge on”. In addition, elaborating finding related with Kiribati culture, in term of respect to the elders and feeding the food leftover for children may help in understanding some of the factors contributing to malnourishment in children, in particular if the food leftover were low quality in terms of nutritional values (e.g., no protein or iron source as red meat or chicken).

I have read this submission. I believe that I have an appropriate level of expertise to confirm that it is of an acceptable scientific standard, however I have significant reservations, as outlined above.

# Peer review: Reviewer response for version 1

**DOI:** 10.5256/f1000research.19390.r43343

**Published:** 2019-02-27

**Authors:** Sari Andajani

**Affiliations:** 1 School of Public Health and Psychosocial Studies, Auckland University of Technology, Auckland, New Zealand

This paper is an original work. The study rationale is sound, the underdeveloped introduction has made it hard for the reviewers to see how this study differ to the previous ones. As findings and discussion are suggestive of the use of Transtheoretical Theory, it needs to be included in the introduction chapter. Findings are presented as series of quotes and at a few points were confusing for readers. I suggest rework of the finding sections, and give signposting to each section. For example for Behavioural factor, states clearly that behaviour factors including mothers' time restriction, conflict of interest, or not keeping up scheduled immunization. Very interesting and specific cultural context was presented in the finding and discussion, unfortunately it has not been described earlier in the study sample. I suggest to add a small Pará on participants context explaining who there are, family, structure, and other relevant context which may support or hinder immunization. A couple of notes on the methods section re 'saturation" nature, purpose of saturation shall be operationalised in a way that is consistent with the research question and theoretical position (in this case seems like the Transtheoretical theory).

 Re: Inter rater reliability to establish consistency of findings. In Qualitative research do not make explicit this concept, however implicitly they can provide descriptions of the procedures for carrying out the analysis. Needs to be referenced and mentioned, saturation to which study question?

 Re: inter-rater reliability, in a qualitative research it is implicitly mention as a process of which are followed by both of the authors. For example meetings of two researchers' to discuss and negotiate agreements and disagreements about coding. Or codings were brainstormed to put the first order statement from respondents and agree them.

I have read this submission. I believe that I have an appropriate level of expertise to confirm that it is of an acceptable scientific standard, however I have significant reservations, as outlined above.

# Peer review: Reviewer response for version 1

**DOI:** 10.5256/f1000research.19390.r43341

**Published:** 2019-02-18

**Authors:** John F. Smith

**Affiliations:** 1 Postgraduate Tropical Medicine Program, Faculty of Medicine, Khon Kaen University, Khon Kaen, Thailand

This modest research project is on a topical and critical issue for many in a wide range of developing countries, effecting not only the developmental trajectory of individual children themselves but also their potential future contributions to their societies.

 The paper is clearly written and very readable throughout. The Method section is clear and replicable.

 Themes from qualitative data analysis are succintly expressed in the Results.  

 The Introduction emphasizes the mother's primary responsibility for care/feeding of children and identifying maternal KAPs in respect to child malnourishment. Not downplaying this, but there are also other broader system factors (determinants) that often influence mothers' health/capacity to provide nourishment for small children, e.g., work/life balance for the mother--does she, by necessity, have to leave the infant in care of others while working during the day?, food security? quality and quantity of good food for the mother's to underpin quality breastfeeding, economic factors impinging on access to good food for the mother etc. Some of these are alluded to in the results under later themes.  

 There appears to have been little in the FGD process to prompt for understandings of these broader social/economic/cultural variables role in malnourishment. Some allusion to these does come up in the  Discussion section but study would have been stronger if these addressed directly via FGD data collection phase. KAP studies typically produce recommendations for strengthening individual's knowledge, behaviour, practices etc via health education, literacy interventions, decontextualised from important broader social/economic, cultural structural variables outside any one individual's capacity to change and that require different intervention approaches. A broader health development/social determinants - driven approach may be needed rather than focusing on individuals skills knowledge etc.

 Notwithstanding the above still a useful, albeit modest, study on this important topic.

I have read this submission. I believe that I have an appropriate level of expertise to confirm that it is of an acceptable scientific standard.
